# Chewing, Stress-Related Diseases, and Brain Function

**DOI:** 10.1155/2015/412493

**Published:** 2015-05-20

**Authors:** Kin-ya Kubo, Huayue Chen, Xiaolin Zhou, Jian-Hua Liu, Olivier Darbin

**Affiliations:** ^1^Seijoh University Graduate School of Health Care Studies, 2-172 Fukinodai, Tokai, Aichi 476-8588, Japan; ^2^Department of Anatomy, Gifu University Graduate School of Medicine, 1-1 Yanaido, Gifu 501-1194, Japan; ^3^Center for Brain and Cognitive Sciences and Department of Psychology, Peking University, Beijing 100871, China; ^4^Department of Oral and Maxillofacial Surgery, The First Affiliated Hospital, College of Medicine, Zhejiang University, Hangzhou 310003, China; ^5^Department of Neurology, University of South Alabama, Mobile, AL 36688, USA

While active mastication or chewing is primarily involved in food intake and digestion, it also promotes and preserves general health. Epidemiologic studies indicate that aged individuals with fewer teeth are more likely to develop cognitive dysfunction. Tooth loss is an epidemiologic risk factor for Alzheimer's disease. Abnormal mastication due to tooth loss or occlusal disharmony induces chronic stress, leading to pathologic changes in the hippocampus and deficits in learning and memory. Evidence suggests that chewing effectively sends various types of information to the brain. Chewing activates several brain regions that are essential for cognitive processing, including the hippocampus and prefrontal cortex. In addition, chewing gum before a meal can decrease food intake and help prevent obesity via neural pathways. In this special issue, several research groups report new findings regarding the importance of mastication.

Y. Ono et al. examined the effects of chewing on stress-induced long-term depression and anxiety-related behaviors in adult male rats. They found that chewing ameliorated the development of long-term depression in the hippocampal CA1 region. Chewing may activate the dopaminergic system in the ventral hippocampus to suppress stress-induced anxiogenic behaviors. This study demonstrates that chewing could be an active coping strategy for relieving stress-induced anxiety-like behaviors, which may be involved in the dopaminergic neuronal pathway.

R. A. F. Weijenberg and F. Lobbezoo explored the relationship between mastication, cognition, and pain. Active mastication might improve some measures of cognitive performance, such as working memory and subjective alertness. These effects can partially be explained by cerebral functional specialization. Stress and relief of stress can play an important role in the physiologic mechanisms underlying these effects. Oral habits such as bruxism might have the same effects relieving stress. Active chewing might relieve stress or pain, but long-term engagement in oral habits such as these increases the risk of fatigue, pain, and temporomandibular disorders.

K.-Y. Kubo et al. provide a concise review of the neuronal mechanisms that underlie the interactions between masticatory function and stress-coping behaviors in animals and humans. Chewing or biting as a stress-coping behavior attenuates stress-induced disorders, such as gastric ulcers, and cognitive and psychological impairment in rodents via suppression of stress-induced activation of the hypothalamic-pituitary-adrenal axis and autonomic nervous system. The histaminergic nervous system may also be involved in the chewing-induced attenuation of stress-induced cognitive deficits. In humans, many studies support an association between stress and sleep bruxism. Gum chewing during stress may affect the levels of various stress markers in the saliva and plasma and increase attention, self-rated alertness, and vigilance.

A. Allen and A. P. Smith report a pilot study investigating the effects of chewing gum on mode and cognitive performance both in the laboratory and naturalistic work situations. Their findings revealed that chewing gum during the workday enhanced productivity and reduced cognitive problems. Thus, chewing gum can attenuate reductions in alertness and enhance worker performance.

T. Otsuka et al. describe a study using functional near-infrared spectroscopy to evaluate the effects of mandibular retrusive deviation on prefrontal cortex activation. Activation of the prefrontal cortex was significantly greater during clenching in the mandibular retrusive condition than during clenching in the control condition. This study demonstrates that functional near-infrared spectroscopy can be used to objectively evaluate the occlusal condition based on activity in the prefrontal cortex.

Y. Hirano and M. Onozuka present a systematic review of 151 published reports on the relationship between chewing and attention. More than half of the reports indicate that chewing has positive effects on attention, especially sustained attention. The effects seem to coincide with an improvement in mood and are influenced by time-on-task.

Y. Ono et al. focused on prefrontal brain activity based on functional near-infrared spectroscopy, a visual analogue scale, and the hemodynamic response. They found that increased hemodynamic responses in the prefrontal area reflect the top-down control of attention and/or self-regulation against uncomfortable somatosensory input, which is a potential marker of detecting the subjective of occlusal discomfort.

In summary, the papers in this special issue highlight several important research strategies for investigating relationship between chewing, stress-related disorders, and brain function. Findings from these studies will greatly contribute to our understanding of the physiologic mechanisms underlying stress-related disorders and chewing as a stress-coping behavior. As the editors of this special issue, we hope that the readers will find these articles interesting, informative, and inspiring.

*Kin-ya Kubo* *Huayue Chen* *Xiaolin Zhou* *Jian-Hua Liu* *Olivier Darbin*

